# Validity of the Yale Food Addiction Scale for Children (YFAS-C): Classical test theory and item response theory of the Persian YFAS-C

**DOI:** 10.1007/s40519-020-00956-x

**Published:** 2020-07-16

**Authors:** Chung-Ying Lin, Vida Imani, Mark D. Griffiths, Amir H. Pakpour

**Affiliations:** 1 Department of Rehabilitation Sciences, Hong Kong Polytechnic University, Hung Hom, Hong Kong; 2 Pediatric Health Research Center, Tabriz University of Medical Sciences, Tabriz, Iran; 3 International Gaming Research Unit, Psychology Department, Nottingham Trent University, Nottingham, UK; 4 Social Determinants of Health Research Center, Research Institute for Prevention of Non-Communicable Diseases, Qazvin University of Medical Sciences, Shahid Bahounar BLV, 3419759811 Qazvin, Iran; 5 Department of Nursing, School of Health and Welfare, Jönköping University, Jönköping, Sweden

**Keywords:** Adolescence, Eating behavior, Eating disorders, Food addiction, Obesity

## Abstract

**Purpose:**

To examine whether the child/adolescent version of the Yale Food Addiction Scale (YFAS-C) is valid to assess the Iranian adolescents who are overweight.

**Methods:**

After using an internationally standardized method to translate the YFAS-C into Persian, 1186 overweight/obese adolescents aged between 13 and 18 years participated in the present study [666 males; mean age = 15.5 (SD = 1.9) years; zBMI = 2.5 (1.0) kg/m^2^]. All the participants completed the Persian YFAS-C alongside Persian versions of the following scales: Eating Disorder Examination Questionnaire (EDEQ), Clinical Impairment Assessment (CIA), Binge Eating Scale (BES), Eating Attitudes Test (EAT-26), and Depression, Anxiety, Stress Scale (DASS-21).

**Results:**

At the scale level, confirmatory factor analysis verified the single-factor structure of the Persian YFAS-C. Additionally, the Persian YFAS-C had promising properties regarding internal consistency (KR20 = 0.81), test–retest reliability (intraclass correlation coefficient = 0.83), separation reliability (person separation reliability = 0.77; item separation reliability = 0.98), and separation index (person separation index = 2.04; item separation index = 8.01). At the item level, all items had satisfactory properties in factor loadings, corrected item-total correlation, test–retest reliability, and infit and outfit mean square. Moreover, no substantial differential item functioning (DIF) was found concerning gender (male vs. female) or weight status (overweight vs. obesity). Significant and moderate correlations were found between the Persian YFAS-C and other psychometric scales assessing eating symptomatology and general psychopathology (*r* = 0.352 to 0.484).

**Conclusion:**

The Persian YFAS-C is a valid instrument that assists healthcare providers in assessing food addiction among Iranian adolescents.

**Level of evidence:**

Level V, cross-sectional descriptive study.

## Introduction

The fourth and fifth editions of the Diagnostic and Statistical Manual of Mental Disorders (DSM-IV, DSM-5) have both defined addiction using clear diagnostic criteria [1, 2]. However, food addiction (FA) has not been formally recognized despite increasing interest and empirical evidence in the condition. Animal models have demonstrated that it is possible to develop addictive-like behaviors relating to specific foods, especially those high in sugar and fat [3]. Neuroimaging studies on human beings have demonstrated similar findings to those found in animal studies. The neuronal circuits activated by drugs in addiction are similar to the neuronal circuits activated by hyper-palatable food among obese individuals, and both circuits are modulated by dopamine [4, 5]. Therefore, an in-depth understanding of FA is needed for healthcare providers to assist such a population in overcoming this specific type of addiction and its impacts on related disorders (e.g., binge eating) or psychological impairment (e.g., depression).

Several studies have examined the prevalence of FA in different populations using the criteria for substance dependence in the DSM-IV [e.g., 6–8, 11]. Moreover, Gearhardt et al. [9] developed the Yale Food Addiction Scale (YFAS) for assessing FA based on the diagnostic criteria for substance dependence in the DSM-IV. More recently, a child/adolescent version of the YFAS (YFAS-C) was developed and validated [10]. With the development of YFAS and YFAS-C, studies concerning FA can be further empirically investigated.

However, the FA studies on children or adolescents, especially in the large Persian-speaking populations (~ 110 million people are native Persian speakers across Iran, Pakistan, Tajikistan, and Afghanistan) [12], are lacking. A major reason for the dearth of FA studies among Persian-speaking children and adolescents is the lack of a validated instrument for usage. Although one recent Iranian study translated the YFAS-C and applied the Persian YFAS-C to 222 elementary school students to assess FA [11], the study did not provide any psychometric evidence for the robustness translated YFAS-C. Researchers and healthcare providers may, therefore, hesitate to use the Persian YFAS-C because the psychometric properties are not known. Consequently, there is an urgent need to validate the Persian YFAS-C and present its psychometric properties using rigorous testing methods.

Two testing theories (classical test theory [CTT] and modern test theory) with different features in assessing the psychometric properties are warranted in examining the Persian YFAS-C. To the best of the present authors’ knowledge, most studies examining the validity of YFAS-C (and YFAS) have only used CTT and no studies have ever utilized modern test theory [8–10, 13, 14]. Modern test theory, such as the item response theory (IRT) model, uses the probability to convert the responses in a psychometric scale into an additive score (i.e., logit) as well as providing the psychometric properties of an instrument in a sample-free pattern [15, 16]. Consequently, the psychometric evidence derived from using IRT is not heavily influenced by sample characteristics, while psychometric evidence derived from CTT is [15, 16]. Therefore, using both theories to examine the psychometric properties of the Persian YFAS-C may integrate the current validity evidence from traditional validity methods in an understudied field [17]. More specifically, the CTT findings in the present study can help corroborate previous evidence testing the psychometric properties of YFAS-C, and findings utilizing modern test theory can provide an enhanced perspective concerning the YFAS-C’s psychometric properties.

The aims of the present study included the following: (i) to translate the YFAS-C into Persian and provide robust validity testing (including CTT and IRT models) of the Persian YFAS-C using a community sample of adolescents who were overweight/obese (OW/OB) in high schools [10]; (ii) to examine the prevalence of FA among the studied adolescents; and (iii) to examine the how Persian YFAS-C score was associated with eating symptomatology (using the Eating Disorder Examination Questionnaire, Clinical Impairment Assessment, Binge Eating Scale, and Eating Attitudes Test) and general psychopathology (using the Depression, Anxiety, Stress Scale).

## Methods

### Translation procedure

The YFAS-C was recently translated into Persian without any validity testing [11]. Therefore, the present study carried out an independent translation of the YFAS-C to ensure robust linguistic equivalency. More specifically, the original YFAS-C was translated according to standardized international guidelines [18, 19] incorporating the following steps. First, the YFAS-C was translated from English to Persian by two bilingual translators who were native Persian speakers. The two translators conducted the translations independently and then synthesized the two translated versions into an interim Persian version. Second, the interim Persian version was translated back into English by two native English speakers who were fluent in both English and Persian. Both back translators conducted the translations independently and had no knowledge of the original English YFAS-C prior to translation. Third, an expert panel including a psychiatrist, nurses, nutritionist, psychologist, and a psychometrician investigated the aspects of cross-cultural equivalency for all the translated YFAS-C items and the original YFAS-C items. Following this, a pre-final version of the Persian YFAS-C was generated and piloted among 36 participants to ensure its readability.

### Participants and process

Between September 2018 and April 2019, a total of 1660 OW/OB adolescents were approached by trained research staff from 20 high schools in Qazvin, Iran. A total of 1189 agreed to participate (response rate of 71.6%). The inclusion criteria were that participants had to (i) be aged between 13 and 18 years (i.e., the definition from Medical Subject Headings that an adolescent is aged between 13 and 18 years [https://www.ncbi.nlm.nih.gov/mesh/68000293]), (ii) have a diagnosis of OW/OB (i.e., body mass index [BMI] ≥ 85th percentile for age and gender) according to the anthropometric parameters (i.e., weight, height, and BMI), and (iii) have parental consent to participate. The exclusion criteria were (i) being pregnant and (ii) having a cognitive impairment. Written informed parental consent and student consent was provided by all participants. The study was approved by the ethics committee of the Qazvin University of Medical Sciences (IR.QUMS.REC.1398.320).

### Measures

#### Yale Food Addiction Scale for Children (YFAS-C)

The YFAS-C comprises 25 items that assess food addiction among the pediatric population and was modified from the adult version (i.e., Yale Food Addiction Scale; YFAS; [9]). The YFAS-C items correspond to seven criteria based on those for substance-used disorders in the fourth (text revised) edition Diagnostic and Statistical Manual of Mental Disorders (DSM-IV-TR; [1]). Three YFAS-C items (Items 19, 20, and 24) are not used for testing the seven criteria but are primers for other questions [10] (see Table 2 regarding the seven criteria items). Items 1 to 18 are rated on a 5-point Likert scale (0 = never; 4 = always) and Items 19 to 25 are rated on a dichotomous (0 = no; 1 = yes) scale. All the items rated on the 5-point scale can be converted into a dichotomous scale, where 0 = no and 1 = yes, according to specific scoring thresholds for each item [20]. A criterion (given up activities; persistent desire; activity to obtain, use, recover; tolerance; inability to cut down; withdrawal; or large amount of time spent) is met if at least one item of each criterion is scored as one. Consequently, two scoring versions can be generated: a symptom count scoring version (ranging between 0 and 7) and a diagnostic scoring version (having three or more criteria met in addition to having a clinically significant impairment or distress) [20, 21]. The internal consistency of the YFAS-C is good (KR-20 = 0.82) and the construct validity of the YFAS-C is supported by confirmatory factor analysis [22].

#### Anthropometric measures

Several research assistants measured the anthropometrics of the participants (including adolescents and their parents) in a classroom. More specifically, a stadiometer (SECA Model 207, SECA, Hamburg, Germany) was used to measure heights to the nearest 0.1 cm (participants with shoes removed); a calibrated digital scale (SECA 888 Digital Scale, SECA, Hamburg, Germany) was used to measure weights to the nearest 0.1 kg (participants with light clothes). Using height and weight, a BMI was calculated and a *Z* score of BMI (z-BMI) was further calculated for all participants [23].

#### Bioelectrical impedance analysis (BIA)

The BIA was measured using a bioimpedence analyzer (InBody 230, Biospace, Seoul, South Korea) which obtained body composition in fat percentage and muscle mass.

#### Eating Disorder Examination Questionnaire (EDEQ)

The EDEQ comprises 28 items that assess the attitudes, feelings, and behaviors concerning eating and body image of an individual [24]. All items are rated on a 7-point Likert scale (0 = never; 6 = every day). A higher score of EDEQ indicates a more severe eating disorder [25]. The internal consistency of the Persian EDEQ is excellent (*α* = 0.91) [26]. The internal consistency of the Persian EDEQ in the present study was good (*α* = 0.79).

#### Clinical impairment assessment (CIA)

The CIA comprises 16 items that assess psychosocial impairment due to the presence of an eating disorder. All items are rated on a 4-point Likert scale (0 = not at all; 3 = a lot). A higher score on the CIA indicates more severe psychosocial impairment [27]. The internal consistency of the Persian CIA is excellent (*α* = 0.93) [26]. The internal consistency of the Persian CIA in the present study was good (*α* = 0.78).

#### Binge Eating Scale (BES)

The BES comprises 16 items that assess whether an individual has binge eating problems. All items contain three or four statements and individuals choose the one that describes them the best. The BES total score ranges between 0 and 46, and a higher score indicates higher tendency for binge eating [28]. The internal consistency of Persian BES is very good (*α* = 0.85) [29]. The internal consistency of the Persian BES in the present study was very good (*α* = 0.84).

#### Eating Attitudes Test (EAT-26)

The EAT-26 comprises 26 items that assess the symptoms and concerns of eating disorders. All items are first rated on a 6-point Likert scale (0 = never; 5 = always) and then converted into a 4-point scale (0 = never, rarely and sometimes; 1 = often; 2 = usually; 3 = always). A higher score on the EAT-26 indicates a higher level of eating disturbance [30–32]. The internal consistency of the Persian EAT-26 is adequate to excellent (*α* = 0.61 to 0.92) [33]. The internal consistency of the Persian EAT-26 in the present study was very good (*α* = 0.81).

#### Depression, Anxiety, Stress Scale (DASS-21)

The DASS-21 comprises 21 items that assess three subtypes of psychological distress (depression, anxiety, and stress). The 21 items correspond to the three psychological distresses with seven items for each type. All items are rated on a 4-point Likert scale (0 = did not apply to me at all, never; 3 = applied to me very much, or most of the time, almost always), and three domain scores (each ranging between 0 and 21) can be computed. A higher domain score indicates a higher level of the psychological distress on that domain [34]. The internal consistency of Persian DASS-21 is very good to excellent (*α* = 0.84 to 0.91; [35, 36]) and its convergent validity is promising [35]. The internal consistency of the Persian DASS-21 in the present study was very good (*α* = 0.86).

### Data analysis

In the reliability and validity testing that utilized CTT, the following statistics were performed: confirmatory factor analysis (CFA), internal consistency using Kuder–Richardson Formula 20 (KR20), and test–retest reliability using intraclass correlation coefficient (ICC). For CFA, the hypothesized model is a first-order structure with 22 YFAS-C items (using dichotomized scores) embedded in a single latent construct (i.e., food addiction), and the data-model fit is excellent if comparative fit index (CFI) and Tucker–Lewis index (TLI) are > 0.95 [37]; root-mean square error of approximation (RMSEA) and standardized root mean square residual (SRMR) are < 0.06 [37]. Multi-group CFA was further conducted to understand whether the Persian YFAS-C has structural and measurement invariance across gender (male vs. female) or across weight status (overweight vs. obesity). When the measurement invariance is supported, data from different groups (e.g., males and females) are organized according to the same factor structure and make the group comparison meaningful [38–41]. The measurement invariance is supported by ∆CFI > − 0.01, ∆SRMR < 0.01, and ∆RMSEA < 0.015 in the nested models of configural model, model with loadings constrained, and model with loadings and intercepts constrained [42]. For the internal consistency, the KR20 > 0.7 is recommended; and for test–retest reliability, the ICC > 0.4 is recommended.

In the reliability and validity testing using the item response theory model (a partial credit model was used for 22 YFAS-C items), the following statistics were performed: the information-weighted mean square (infit MnSq), outlier sensitive MnSq (outfit MnSq), differential item functioning (DIF) across gender, and DIF across weight status (OB vs*.* OW), item separation reliability, person separation reliability, item separation index, and person separation index. The recommended cutoffs are between 0.5 and 1.5 for infit and outfit MnSq [43, 44]; < 0.5 for DIF contrast [43, 44]; > 0.7 for item and person separation reliability; and > 2 for item and person separation index.

In addition, the endorsement rates for the YFAS-C symptoms and clinical thresholds were computed by summing up many participants met or did not meet a diagnostic criterion. Also, the associations between the YFAS-C and assessments on eating symptomatology and general psychopathology were analyzed using Pearson’s correlation coefficient.

## Results

The mean age of the participants was 15.5 years (SD = 1.9) years (*n* = 1189). Slightly more than half of the participants were males (*n* = 666; 56.0%). On average, their BMI was 28.4 (SD = 4.2) and z-BMI was 2.5 (SD = 1.0). Their scores on the psychometric scales and their parents’ demographics are presented in Table 1.

**Table 1:** Participants’ characteristics (*N* = 1189)

	Mean (SD) or *n* (%)	Range
Age (year)	15.5 (1.9)	13–19
Gender (male)	666 (56.0)	–
Fathers’ educational year	7.9 (3.5)	0–18
Mothers’ educational year	6.6 (3.8)	0–18
BMI (kg/m^2^)	28.4 (4.2)	25.9–48.6
BMI (*z* score)	2.5 (1.0)	1.6–2.84
Mother BMI (kg/m^2^)	36.5 (4.4)	26.1–50.9
Father BMI (kg/m^2^)	34.3 (4.2)	26.9–54.7
Score in depression^a^	8.1 (4.1)	6.0–27.3
Score in anxiety^a^	8.8 (4.6)	5.0–30.70
Score in stress^a^	8.0 (5.1)	11.1–29.0
Body fat percentage bioelectrical impedance analysis (BIA)	33.4 (7.1)	25.0–40.1
Score in Eating Disorder Examination Questionnaire (EDEQ)	1.78 (0.99)	0.21–0.6.0
Score in Clinical Impairment Assessment (CIA)	7.53 (6.22)	0–48
Score in Binge Eating Scale (BES)	12.68 (5.9)	0–46
Score in Eating Attitudes Test (EAT-26)	24.3 (11.0)	0–78
Score in Food addiction symptoms^b^	2.69 (1.64)	0–7

^a^Measured using Depression, Anxiety, Stress Scale

^b^Based on the symptom count version of the Yale Food Addiction Scale

Table 2 illustrates the mean, SD, skewness, and kurtosis of all the Persian YFAS-C items. In general, all the items were not extremely skewed. Table 3 further demonstrates the item properties in the factor loadings (0.40–0.70), corrected item-total correlation (0.40–0.64), test–retest reliability (0.70–0.94), infit MnSq (0.75–1.26), outfit MnSq (0.52–1.55), item difficulty (− 3.02–1.39), item discrimination (0.35–1.34), and DIF across gender (− 0.47–0.44) and weight status (− 0.46–0.49). More specifically, all items have acceptable properties, except for the outfit MnSq for Item 17 (1.55).

**Table 2:** Item properties of the Yale Food Addiction Scale for Children

Diagnostic criteria Item #	Mean (SD)	Skewness	Kurtosis
Substance is taken in larger amount and for longer period than intended			
#1 When I start eating, I find it hard to stop	0.10 (0.09)	2.72	3.39
#2 I eat food even when I am not hungry	0.11 (0.11)	2.65	3.04
#3 I eat until my stomach hurts or I feel sick	0.15 (0.36)	1.98	1.94
Persistent desire or repeated unsuccessful attempts to quit			
#4 I worry about eating too much food	0.11 (0.31)	2.48	4.16
#17 I want to cut down or stop eating certain foods	0.03 (0.05)	3.85	6.99
#18 How often do you try to cut down on certain foods?	0.04 (0.07)	3.94	7.11
#25 I am able to cut down on certain foods	0.17 (0.27)	1.72	0.96
Much time is spent to obtain and to use the substance or to recover from its effects			
#5 I feel tried a lot because I eat too much	0.23 (0.42)	1.27	− 0.39
#6 I eat food all day long	0.06 (0.23)	3.69	5.63
#7 If I cannot find a food I want, I will try hard to get it (ask a friend to get it for me, find a vending machine, and sneak food when people are not looking)	0.14 (0.24)	2.10	2.43
Important social, occupational, or recreational activities are given up or reduced because of substance use			
#8 I eat food rather than do other things I like (e.g., play and hang out with friends)	0.12 (0.21)	2.48	4.11
#9 I eat so much that I feel bad afterward. I feel so bad that I do not do things I like (e.g., play and hang out with friends)	0.05 (0.20)	4.32	5.72
#10 I avoid places that have a lot of food, because I might eat too much	0.12 (0.31)	2.43	3.89
#11 I avoid places where I cannot eat the food I want	0.10 (0.29)	2.70	5.30
The substance is continued to be used despite knowledge of adverse consequences			
#21 I eat in the same way even though it is causing problems	0.44 (0.39)	0.25	− 1.94
Tolerance			
#22 I need to eat more to get the good feelings I want (feel happy, calm, and relaxed)	0.55 (0.49)	− 0.22	− 1.95
#23 When I eat the same amount of food, I do not feel good the way I used to (feel happy, calm, and relaxed)	0.53 (0.41)	− 0.13	− 1.38
Characteristic withdrawal symptoms; substance taken to relieve withdrawal			
#12 When I do not eat certain foods, I feel upset or sick	0.05 (0.03)	1.11	3.98
#13 I eat certain foods to stop from feeling upset or sick	0.08 (0.27)	3.06	4.38
#14 When I cut down or stop eating certain foods, I crave them a lot more	0.10 (0.29)	2.74	5.56
Use causes clinically significant impairment or distress			
#15 The way I eat makes me a really unhappy	0.10 (0.20)	2.66	3.12
#16 The way I eat causes me problems (problems at school, with my parents, and with my friends)	0.05 (0.21)	2.15	5.27

**Table 3:** Psychometric properties of the Yale Food Addiction Scale for children in item levels

Item #	Analyses from classical test theory			Analyses from item response theory					
	Factor loading^a^	Item total correlation	Test–retest reliability^b^	Infit MnSq	Outfit MnSq	Difficulty	Discrimination	DIF contrast across gender^c,d^	DIF contrast across weight status^c,e^
#1	0.41	0.49	0.73	1.03	0.88	− 0.11	1.17	− 0.17	0.31
#2	0.53	0.50	0.78	0.88	0.73	− 0.16	1.14	0.21	0.49
#3	0.51	0.46	0.74	0.95	0.96	− 0.75	0.96	0.01	0.33
#4	0.44	0.42	0.83	1.12	1.04	− 0.30	0.89	0.24	0.12
#5	0.43	0.45	0.84	0.94	0.85	− 1.52	0.87	0.43	0.24
#6	0.53	0.52	0.88	0.96	0.59	0.57	1.00	− 0.23	0.33
#7	0.63	0.46	0.92	1.08	1.29	− 0.63	0.81	0.27	− 0.30
#8	0.69	0.60	0.76	0.89	0.74	− 0.30	1.08	0.19	− 0.22
#9	0.70	0.64	0.80	0.75	0.58	0.93	1.13	0.44	0.21
#10	0.51	0.54	0.86	0.96	0.68	− 0.35	1.03	0.16	0.45
#11	0.55	0.54	0.94	0.96	0.82	− 0.12	1.01	0.43	0.49
#12	0.69	0.58	0.88	0.85	0.52	0.82	1.09	− 0.47	0.23
#13	0.70	0.57	0.72	0.92	0.70	0.15	1.05	− 0.27	0.41
#14	0.68	0.59	0.70	0.91	0.64	− 0.08	1.17	− 0.39	− 0.22
#15	0.66	0.60	0.76	0.89	0.58	− 0.15	1.08	0.34	− 0.43
#16	0.64	0.57	0.72	0.88	0.65	0.84	1.03	0.09	− 0.14
#17	0.49	0.41	0.82	1.10	1.55	1.35	1.17	0.41	− 0.42
#18	0.46	0.40	0.75	1.09	1.02	1.39	1.19	0.01	− 0.34
#21	0.40	0.40	0.73	1.26	1.49	− 2.90	1.15	0.43	− 0.39
#22	0.41	0.42	0.79	1.16	1.47	− 3.02	1.34	0.21	− 0.46
#23	0.44	0.48	0.82	1.20	1.17	0.19	1.31	− 0.24	− 0.17
#25	0.57	0.41	0.77	1.24	1.33	− 1.01	0.35	− 0.41	0.38

*MnSq *mean square error, *DIF *differential item functioning

^a^Based on confirmatory factor analysis

^b^Using intraclass correlation coefficient

^c^DIF contrast > 0.5 indicates substantial DIF

^d^DIF contrast across gender = difficulty for females − difficulty for males

^e^DIF contrast across weight status = difficulty for participants with obesity − difficulty for participants with overweight

The one-factor structure of the Persian YFAS-C was supported by the CFA fit indices (CFI = 0.93, TLI = 0.91, RMSEA = 0.048, and SRMR = 0.040) although CFI and TLI were slightly lower than the cutoffs. Also, internal consistency (KR20 = 0.81) and the test–retest reliability (ICC = 0.83) of the entire Persian YFAS-C were satisfactory (Table 4). Moreover, multi-group CFA showed that the Persian YFAS-C was measurement invariant across both gender and weight status (Table 5). The IRT analysis indicated that the Persian YFAS-C was unidimensional (item separation reliability = 0.98; item separation index = 8.01; person separation reliability = 0.77; person separation index = 2.04) (Table 4).

**Table 4:** Psychometric properties of Yale Food Addiction Scale for children (YFAS-C) in scale level

Psychometric testing	YFAS-C	Suggested cutoff
Internal consistency (Kuder–Richardson Formula 20)	0.81	> 0.7
Confirmatory factor analysis		
*χ*^2^ (*df*)	775.39 (209)*	Non-significant
Comparative fit index	0.93	> 0.9
Tucker–Lewis index	0.91	> 0.9
Root-mean square error of approximation	0.048	< 0.08
Standardized root mean square residual	0.040	< 0.08
Item separation reliability from item response theory	0.98	> 0.7
Item separation index from item response theory	8.01	> 2
Person separation reliability from item response theory	0.77	> 0.7
Person separation index from item response theory	2.04	> 2
Test–retest reliability by intraclass correlation coefficient	0.83	> 0.4

*p < 0.001

**Table 5:** Measurement invariance across gender and across weight status through confirmatory factor analysis

Model and comparisons	Fit statistics							
	*χ*^2^ (df)	∆*χ*^2^ (∆df)	CFI	∆CFI	SRMR	∆SRMR	RMSEA	∆RMSEA
Gender (male vs*.* female)								
M1: Configural	894.938 (418)*		0.975		0.028		0.034	
M2: Plus all loadings constrained	922.462 (440)*		0.978		0.024		0.032	
M3: Plus all intercepts constrained	951.370 (462)*		0.980		0.020		0.031	
M2−M1		27.524 (22)		0.003		− 0.004		− 0.002
M3−M2		28.908 (22)		0.002		− 0.002		− 0.001
Weight status (overweight vs*.* obesity)								
M1: Configural	1039.148 (418)*		0.971		0.032		0.038	
M2: Plus all loadings constrained	1063.254 (440)*		0.973		0.030		0.038	
M3: Plus all intercepts constrained^b^	1091.611 (462)*		0.976		0.028		0.035	
M2−M1		497.66 (22)		− 0.032		0.027		0.035
M3−M2		28.357 (22)		− 0.003		− 0.002		− 0.003

M1 = Model 1, a configural model; M2 = Model 2, a model based on M1 with all factor loadings constrained being equal across groups; M3 = Mode 3, a model based on M2 or M2P with all item intercepts constrained being equal across groups

*CFI *comparative fit index, *SRMR *standardized root mean square residual, *RMSEA *root mean square error of approximation

*p < 0.05

Table 6 demonstrates how many participants met each diagnostic criterion of the Persian YFAS-C. More than half of the participants met the diagnostic criterion of “inability to cut down” (54.6%) and only few participants had a “persistent desire” (7.1%). As a result, slightly more than one-third of the participants (*n* = 429; 36.1%) had met at least three diagnostic criteria. The prevalence of FA was 12.1% in the adolescent sample. Moreover, the Persian YFAS-C was significantly associated with eating symptomatology and moderately associated with general psychopathology: *r* = 0.412 with CIA; 0.439 with EAT-26; 0.396 with EQED; 0.484 with BES; 0.384 with depression in the DASS-21; 0.352 with anxiety in the DASS-21; and 0.401 with stress in the DASS-21.

**Table 6:** Endorsement rates for YFAS-C “symptoms” and clinical thresholds

	Met symptom/threshold^a^	Did not meet symptom/threshold^a^
Given up activities	240 (20.2%)	949 (79.8%)
Persistent desire	85 (7.1%)	1104 (92.9%)
Activity to obtain, use, recover	377 (31.7%)	812 (68.3%)
Tolerance	376 (31.6%)	813 (68.4%)
Inability to cut down	649 (54.6%)	540 (45.4%)
Withdrawal	182 (15.3%)	1007 (84.7%)
Large amount of time spent	271 (22.8%)	918 (77.2%)
3 ≥ symptoms^a^	429 (36.1%)	760 (63.9%)
Clinically significant impairment or distress	144 (12.1%)	1045 (87.9%)

^a^Threshold indicates the participant met three or more symptoms from the seven symptoms (given up activities; persistent desire; activity to obtain, use, recover; tolerance; inability to cut down; withdrawal; and large amount of time spent)

## Discussion

Utilizing two major types of testing theory, the present study’s findings verify and support the psychometric robustness of the Persian YFAS-C. The CTT findings are comparable with extant evidence concerning the psychometric properties of YFAS-C and the YFAS. The preliminary psychometric findings in the original development of the YFAS-C reported a marginal value in internal consistency (KR-20 = 0.67) [45]. A similar and marginal value in the internal consistency was also observed in the YFAS-C among African American adolescents (KR-20 = 0.68) [14] and Korean adolescents (KR-20 = 0.69) [13]. Although the internal consistency in the present study (KR-20 = 0.81) was higher than these previous findings, all the evidence regarding internal consistency indicates that the YFAS-C has acceptable reliability. Also, the higher internal consistency shown in the present study can be explained by development maturity. The participants in the present study were aged between 13 and 18 years with a mean age of 15.5 years, which is older (and more mature) than the study participants in other studies which recruited participants aged between (i) 4 and 16 years (mean age = 8.32 years) [45]; 12 and 16 years (mean age = 13.75 years) [14]; 11 and 15 (mean age = 13.74 years) [13]. Another study examining the YFAS-C using an older adolescent sample (aged between 8 and 18 years; mean age = 15.1 years) showed good internal consistency (KR-20 = 0.82) [22]. Moreover, good internal consistency has been demonstrated in most research testing the psychometric properties of the YFAS (i.e., adult version) supporting the notion of developmental maturity. For example, one study reported an internal consistency of 0.71 among people with eating disorder with an average age of 29.3 years [8].

Although the findings regarding the internal consistency of the YFAS-C are somewhat varied, studies assessing the factorial structure of the YFAS-C share the same finding: the YFAS-C has a unidimensional structure. Gearhardt et al. [45] found satisfactory fit indices in their CFA model (CFI = 0.94 and RMSEA = 0.08), and similar findings were reported by Magyar et al. [22] (CFI = 0.896 and RMSEA = 0.0528) and the present study (CFI = 0.93 and RMSEA = 0.048). The psychometric findings reported here further extend the verification of the YFAS-C structure from CFA findings to those derived from IRT models (infit MnSq and outfit MnSq all between 0.5 and 1.5). Given that no prior research has applied the IRT model to examine the psychometric properties of the YFAS-C, no comparison of the IRT results here can be made with the extant literature. However, the IRT findings strengthen the notion of the unidimensional structure of the YFAS-C. Moreover, because IRT models are sample free [12, 15], researchers can be confident that the unidimensional structure of the YFAS-C can be applied to different adolescent populations.

Using the validated Persian YFAS-C, the prevalence of FA in the present sample was 12.1%, which is comparable to other pediatric or adolescent populations, Magyar et al. [22] reported that 8.9% of Hungarian native children and adolescents residing in Baranya area had FA. Schulte et al. [14] reported that 10.1% of African American OW/OB adolescents who were enrolled in a 6-month weight loss intervention program had FA, and Gearhardt et al. [45] reported that 7.2% of American children residing in New Haven community had FA. Therefore, the prevalence of FA is relatively high across different ethnic populations and is, therefore, a public health issue that should not be ignored.

The findings of the present study also echo previous findings that FA is associated with eating symptomatology and psychopathology [8]. Therefore, the presence of FA symptoms appear to be an early sign of problematic eating and psychological distress. From the present study’s findings, the variance of FA-related symptoms had 15.6–23.4% overlap with that of different types of eating disorders. The variance of FA-related symptoms had 12.4–16.1% overlap with that of different types of psychological distress. The overlap between FA-related symptoms, eating disorders, and psychological distress found in the present study is consistent with prior findings [45–47]. Several studies on patients with binge eating disorder have found that more FA-related symptoms are associated with higher levels of negative emotions and more severe eating pathology [45–47]. Therefore, healthcare providers may want to closely monitor the FA symptom of adolescents to prevent them from developing serious health consequences.

There are some limitations in this study that should be taken into consideration when interpreting the findings. First, the recruited adolescents were community-based rather than clinical-based. Therefore, whether our findings can be generalized to adolescents with eating-related disorders is questionable. It will be important to continue the validity testing of the Persian YFAS-C among adolescents who receive treatment for eating (or related) disorders. Second, the associations between FA, eating symptomology, and psychopathology were based on a cross-sectional design. Therefore, the causality cannot be concluded. Future studies using longitudinal or case–control design are, therefore, warranted to further explore the potential causal relationship between FA, eating symptomology, and psychopathology. Third, FA, eating symptomology, and psychopathology were all assessed using self-reports. Therefore, common bias in self-reports, such as social desirability and recall bias, could have confounded the findings.

## Conclusion

Based on the findings of the present study, the Persian YFAS-C is a valid instrument that can assist healthcare providers in assessing FA among Iranian adolescents. The validity and reliability of Persian YFAS-C were verified and supported by rigorous evaluation utilizing two major testing theories (i.e., CTT and IRT models). Additionally, FA was found to be prevalent among Iranian adolescents (12.1%) and was moderately associated with eating symptomatology and psychopathology. Consequently, healthcare providers should not ignore the issue of FA among adolescents.

## What is already known on this subject?

The Yale Food Addiction Scale for Children (YFAS-C) is a commonly used instrument to assess food addiction for children and has been validated using classical test theory (CTT). The psychometric testing using CTT shows that YFAS-C is a promising instrument. However, it is unclear whether the YFAS-C has the same psychometric properties using another assessment theory (i.e., modern test theory) and it is unclear whether the YFAS-C has good properties in its Persian version.

## What does this study add?

The study results indicated that the Persian YFAS-C has strong psychometric properties in both CTT and modern test theory results. With the robust psychometric properties, the Persian YFAS-C can assist clinicians in understanding the level of food addiction for Persian children and adolescents.
